# Proteasome-Mediated Proteolysis of SRSF5 Splicing Factor Intriguingly Co-occurs with SRSF5 mRNA Upregulation during Late Erythroid Differentiation

**DOI:** 10.1371/journal.pone.0059137

**Published:** 2013-03-11

**Authors:** Osman Breig, Faouzi Baklouti

**Affiliations:** "mRNA Metabolism in Normal and Pathological Cells"; Centre de Génétique et de Physiologie Moléculaire et Cellulaire, CNRS UMR, Université Lyon 1, Villeurbanne, France; NIGMS, NIH, United States of America

## Abstract

SR proteins exhibit diverse functions ranging from their role in constitutive and alternative splicing, to virtually all aspects of mRNA metabolism. These findings have attracted growing interest in deciphering the regulatory mechanisms that control the tissue-specific expression of these SR proteins. In this study, we show that SRSF5 protein decreases drastically during erythroid cell differentiation, contrasting with a concomitant upregulation of SRSF5 mRNA level. Proteasome chemical inhibition provided strong evidence that endogenous SRSF5 protein, as well as protein deriving from stably transfected SRSF5 cDNA, are both targeted to proteolysis as the cells undergo terminal differentiation. Consistently, functional experiments show that overexpression of SRSF5 enhances a specific endogenous pre-mRNA splicing event in proliferating cells, but not in differentiating cells, due to proteasome-mediated targeting of both endogenous and transfection-derived SRSF5. Further investigation of the relationship between SRSF5 structure and its post-translation regulation and function, suggested that the RNA recognition motifs of SRSF5 are sufficient to activate pre-mRNA splicing, whereas proteasome-mediated proteolysis of SRSF5 requires the presence of the C-terminal RS domain of the protein. Phosphorylation of SR proteins is a key post-translation regulation that promotes their activity and subcellular availability. We here show that inhibition of the CDC2-like kinase (CLK) family and mutation of the AKT phosphorylation site Ser86 on SRSF5, have no effect on SRSF5 stability. We reasoned that at least AKT and CLK signaling pathways are not involved in proteasome-induced turnover of SRSF5 during late erythroid development.

## Introduction

Serine-arginine-rich (SR) proteins, also called SR splicing factors (SRSFs, [1]) are highly conserved family of regulators of pre-mRNA splicing. All SR protein knockout mice displayed an early embryonic lethal phenotype, thus evidencing the fundamental function of SR proteins in vivo [2]. The recent burst of discoveries has dealt with recurrent somatic alterations, found in myeloid disease, and occurring in multiple genes encoding spliceosomal components or non spliceosomal splicing factors, including SR proteins ([3], [4], and references therein). SR protein structure consists of one or two copies of an RNA-recognition motif (RRM) at the N-terminus, and a domain rich in alternating serine and arginine residues (the RS domain) at the C-terminus [5], [6]. SR proteins play a prominent role in splice site selection [2]; they are believed to interact with exonic splicing enhancers (ESEs) at the pre-mRNA molecule, and recruit other splicing components via their RS domain, to promote 3′ splice site selection by U2AF and 5′ splice site recognition by U1 snRNP [7]. SR proteins also regulate pre-mRNA alternative splicing in a concentration-dependent manner. In fact, they have been shown to antagonize the negative activity of heterogeneous nuclear ribonucleoproteins (hnRNPs) bound to nearby sequences, called exonic splicing silencer (ESS) elements [8]. Recent works have implicated SR proteins as pivotal regulators in virtually all steps of mRNA metabolism, including mRNA export, stability, quality control, and translation [9], [10]. Disruption of these functions may lead to developmental defects or disease [11]. Importantly, the phosphorylation status of SR proteins defines their availability and their activity [12], and links pre-mRNA splicing to extracellular signaling [13]. The RS domain of SR proteins undergoes reversible phosphorylation during spliceosome maturation by several protein kinase families, including the serine/arginine-rich protein kinases (SRPKs), the CDC2-like kinase family (CLKs), and the AKT family [12], [14]

SRSF5, previously called SRp40 [1], is a member of the SR protein family, that has early been identified as a splicing regulator [15]. It is expressed as insulin-induced protein in regenerating liver [15], and as a TGF-β1-induced splicing factor that enhances EDA exon inclusion in fibronectin mature mRNA in chondrocytes [16]. However, SRSF5 is best characterized as a major regulator of Human Immunodeficiency Virus Type 1 (HIV-1) mRNA splicing: it activates a purine-rich ESE within HIV exon 5, which enhances the expression of the *env* gene mRNA [17]. Enzymatic and chemical footprinting experiments led to finely delineate binding sites on SLS2 and SLS3 for SRSF5, among other splicing factors, and helped to better understand the expression activation of the TAT protein, which plays a crucial role in the virus mutiplication [18]. More recently, SRSF5 has been shown to promote HIV-1 Gag translation from unspliced viral RNA [19].

SRSF5 is encoded by a unique gene, *SRSF5*, also named *HRS* and *SFRS5*, located on chromosome 14 in human and chromosome 12 in mouse. This gene is ubiquitously expressed in Mammals, and displays different splicing isoforms, among which long forms with retained introns [15], [20]–[22]. These long forms exhibit stop codons in all three reading frames; in fact, only a protein of 40 kDa has been detected so far.

Little is known regarding SRSF5 expression regulation, and in a more general perspective, regarding the regulation of SR protein family during cell differentiation. In an early study, we have documented a differential expression of several SR proteins during erythroid differentiation (Huang et al. (2000) Blood 96: 592a; abstr.). In the present study, we documented an increase of SRSF5 mRNA accumulation, contrasting with a dramatic decrease of the protein level, during erythroid differentiation. Consistently, we demonstrated a positive effect of SRSF5 on a specific alternative splicing event in proliferating erythroid cells, but not in differentiating cells. We found that SRSF5 downregulation is due to a proteasome-mediated proteolysis during erythroid cell differentiation. Interestingly, we argued that lack of the RS domain of SRSF5 prevents from proteasome degradation, but does not alter the splicing activity of the truncated SRSF5. We further showed that phosphorylation by either the CLK or AKT signaling is not required for regulated turnover of SRSF5 induced by the proteasome.

## Results

### Opposite patterns of expression of SRSF5 mRNA and protein during late erythroid differentiation

Preliminary data have suggested that several SR proteins, including SRSF1, SRSF2 and SRSF7, are upregulated during erythroid differentiation of mouse erythroleukemia (MEL) cells (Huang et al. (2000) Blood 96: 592a; abstr.). To analyze more specifically the pattern of expression of SRSF5, we first estimated the amount of mature mRNA in MEL cells. Also called Friend cells, these cells provide an excellent cell model that has been successfully used for decades to reproduce terminal erythroid differentiation *in vitro* [23]. MEL cells are proerythroblasts arrested in their differentiation at the colony-forming units-erythroid (CFU-E) stage. However, they are able to further differentiate to hemoglobinized cells upon exposure to a chemical inducer, such as dimethylsulfoxide (DMSO) or hexamethylene-bisacetamide (HMBA).

**Figure 1:**
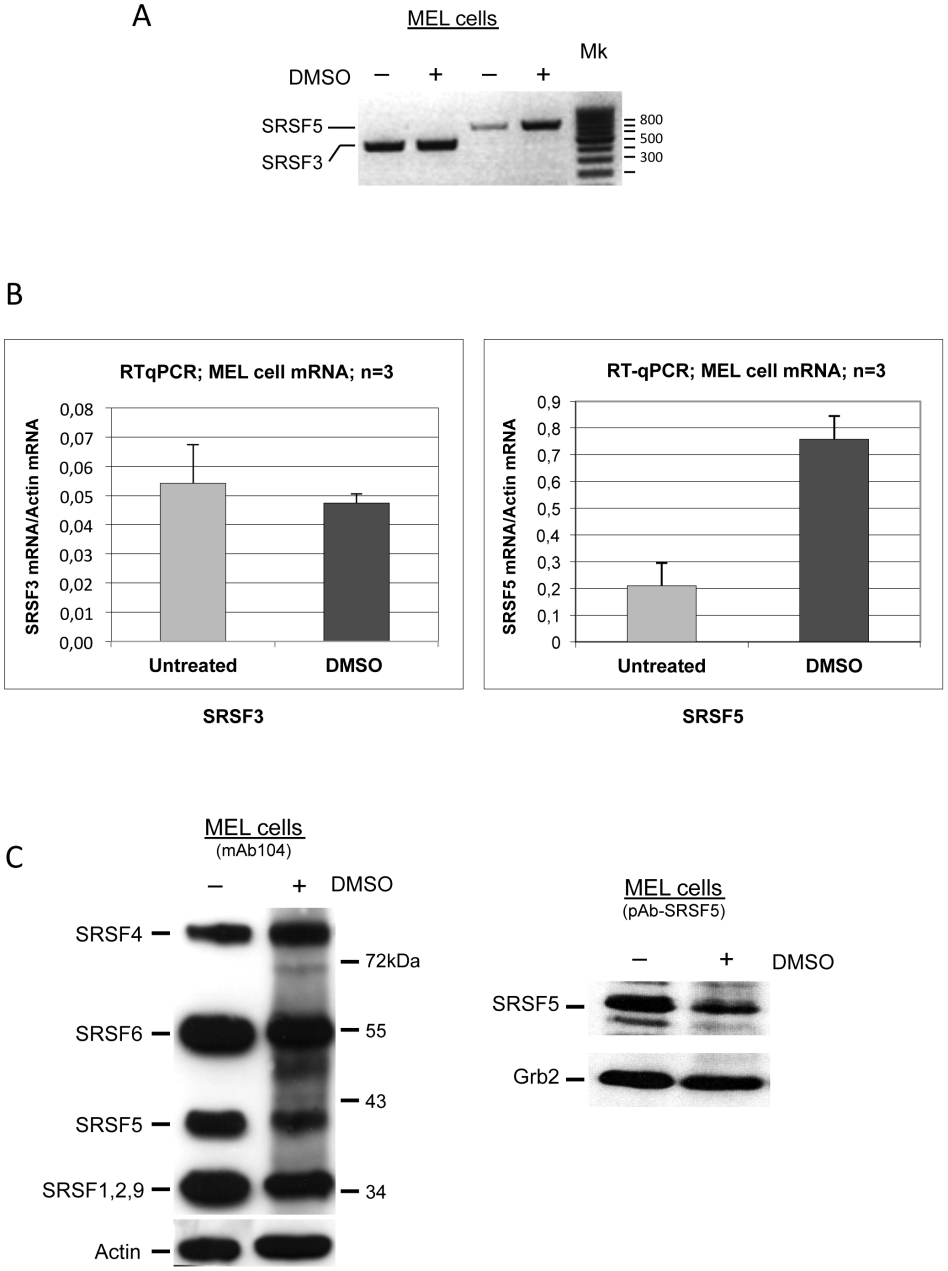
Opposite patterns of expression of SRSF5 mature mRNA and protein during late erythroid differentiation. A. Steady-state mRNA analysis. Cells were cultured in the absence (−) or presence (+) of DMSO for 4 days to trigger erythroid differentiation. SRSF5 and SRSF3 mRNAs were amplified using specific appropriate forward and reverse primers (see text). Mk: size markers.B. Real-time RT-PCR analysis of SRSF5 and SRSF3 mRNAs. Steady-state mRNA levels were normalized with respect to actin mRNA, used as internal control. Cells were cultured in the absence (untreated) or presence (DMSO) of DMSO for 4 days. Note that cell induction to erythroid differentiation led to a more than threefold increase of steady-state levels of SRSF5 mRNA, when compared with uninduced cells, whereas SRSF3 mRNA remained roughly unchanged. C. Immunoblot analysis of SRSF5 expression in MEL cells. Cells were left untreated (−), or treated (+) with 1.8% DMSO for 4 days. SRSF5 was revealed using either mAb104, an antibody that immunoreacts with all the prototypical SR proteins (left panel), or pAb-SRSF5, a specific anti-SRSF5 antibody (right panel). MEL cell induction using DMSO resulted in a decrease of SRSF5 protein signal.Actin and Grb2 were used as controls.

Cells were cultured in the absence or presence of DMSO to induce cell erythroid differentiation. Qualitative and real-time RT-PCR were performed on SRSF5 and SRSF3, another member of the SR protein family for which our preliminary experiments have suggested a constant expression during erythroid differentiation. As shown in Figure 1A and B, both qualitative and real-time RT-PCR experiments consistently revealed that SRSF3 mRNA accumulation was virtually constant during MEL cell differentiation, whereas SRSF5 exhibited a clear increase in mRNA accumulation in differentiating cells, as compared with its level of expression in uninduced cells.

We addressed the possible degradation of SRSF5 mRNA via nonsense-mediated mRNA decay (NMD) mechanism, in proliferating cells, using a previously described approach [24]. No difference was observed in mRNA expression level in cells before and after treatment with caffeine and cyclohexamide (not shown), ruling out a possible modulation of mRNA accumulation by the NMD mechanism.

We next asked whether the protein level reflects the mRNA upregulation. We therefore examined SRSF5 expression in cells induced to erythroid differentiation with DMSO. Immunoblotting analysis of SRSF5 expression was performed using two distinct antibodies, directed either against the phosphoepitopes of prototypical SR protein family members, or specifically against SRSF5 (Figure 1C). This experiment revealed a reduction in SRSF5 protein signal as the cells underwent terminal differentiation.

**Figure 2:**
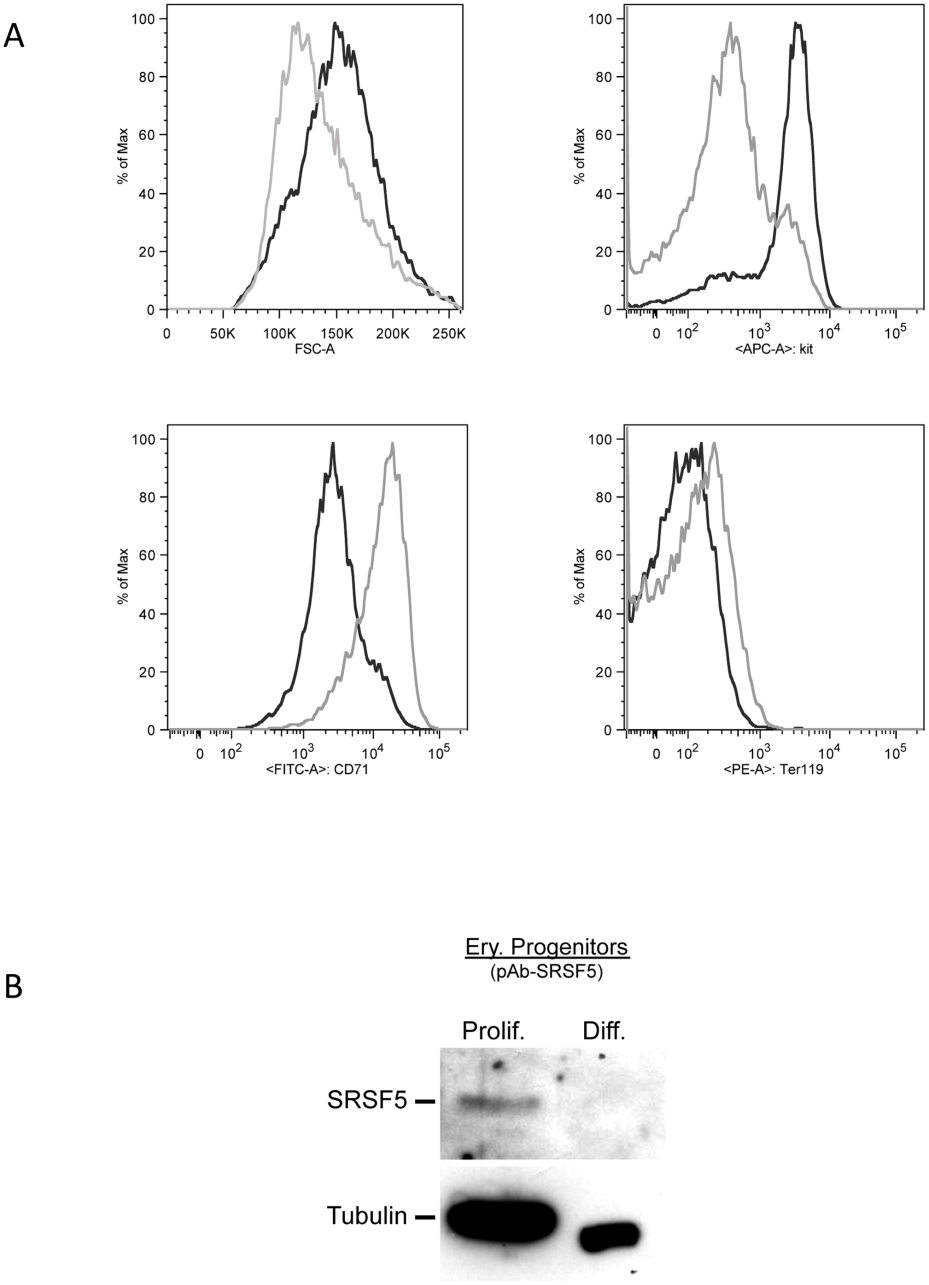
SRSF5 expression in primary ex-vivo erythroid precursors. A. Flow cytometric analysis of proliferating (black line) and differentiating (grey line) cells. In each graph, the intensity of scatter (FSC) or fluorescence (kit (CD117), CD71, Ter119) is plotted on the Y-axis. The number of events (number of cells) is plotted on the X-axis; it is expressed as 10^3^-fold (K) in FSC histogram. Proliferating erythroblasts are large cells (high FSC). They express moderate to high levels of Kit and CD71, and low levels of Ter119 on their cell surface. After 2 days of maturation, these cells display a cell size decrease (low FSC), and cell surface phenotype changes, including Kit decrease and higher levels of CD71 and Ter119.B. Immunoblot analysis of SRSF5 expression in fetal liver-derived erythroblasts. Proteins were collected from proliferating cultured erythroblasts (Prolif.) and 2 days after ex-vivo differentiation (Diff.). Western blot analysis was performed using anti-SRSF5 antibody "pAb-SRSF5". Note that SRSF5 virtually vanishes as the cells differentiate.

To determine whether the observed change in SRSF5 signal is related to the transformed feature of MEL cells or rather reflects a physiologic aspect of erythroid differentiation, we analyzed SRSF5 expression in primary erythroid precursors derived from mouse fetal liver [25]. The proliferating cultures consist primarily of immature erythroblasts, large cells that coexpress Kit, CD71 and low levels of the erythroid-specific marker Ter119. These self-renewing erythroblasts maintain their ability to mature into erythrocytes when cultured in erythroid maturation media (Materials and Methods, [25]). Flow cytometric analyses of the ex-vivo proliferating erythroblasts showed a phenotype similar to primary proerythroblasts in the bone marrow and fetal liver: a high forward scatter parameter (FSC), the cell surface expression of Kit, CD71, and low levels of Ter119 (Figure 2A, [25]). Maturation of cultured erythroblasts is characterized by changes in the cell surface phenotype. Analysis by flow cytometry showed marked down-regulation of Kit and up-regulation of Ter119 and CD71, associated with a decrease in FSC (Figure 2A). SRSF5 expression was analyzed in proliferating erythroblasts, and after 2 days of maturation. Immunoblot analysis showed a decrease in SRSF5 epitopes as the cells differentiated (Figure 2B).

Altogether, these results suggest that, while the steady-state mRNA level increases, the SRSF5 protein exhibits an opposite pattern of expression, due most likely to a downregulation at the protein level, during late erythroid differentiation.

### Exogenous SRSF5 is downregulated in differentiating erythroid cells

**Figure 3:**
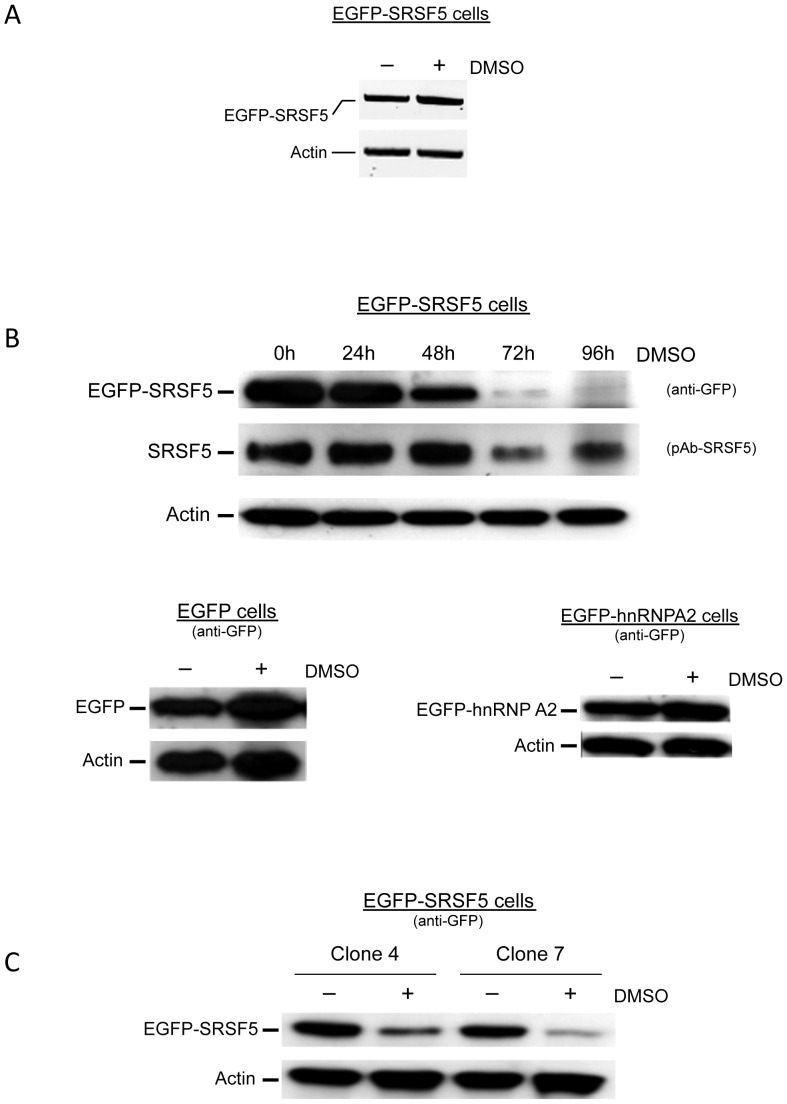
Downregulation of stably expressed SRSF5 protein during erythroid differentiation. A. Recombinant EGFP-SRSF5 mRNA expression. SRSF5 mRNA expressed from the fusion construct EGFP-SRSF5 was amplified from transfected cells using forward primer F8, and reverse primer R8 (Table S1). RNA was extracted from untreated cells or from cells exposed to DMSO-induction for 4 days. Actin mRNA was amplified with primers F6 and R6 (Table S1), and used as control. B. SRSF5 expression during erythroid differentiation. A time course DMSO-induction experiment was performed on MEL cells, transfected with EGFP-SRSF5 construct. Expression of endogenous SRSF5 and fusion EGFP-SRSF5 protein was assessed using anti-SRSF5 or anti-EGFP antibodies, respectively. Expression of control EGFP-containing proteins was also assessed by immunoblotting. These control proteins were obtained from cells transfected with the mock construct EGFP (EGFP cells), or a construct expressing EGFP-hnRNPA2 fusion (EGFP-hnRNPA2 cells), and cultured in the absence (−) or presence (+) of DMSO. C. Downregulation of EGFP-SRSF5 fusion is not clonal. Clones 4 and 7 of MEL cells stably transfected with EGFP-SRSF5 construct were analyzed before (−) or 96 h after (+) DMSO induction. Fusion protein was revealed using anti-GFP antibody.Actin served as an internal control.

We next analyzed in parallel SRSF5 mRNAs and proteins, expressed from stably transfected EGFP-SRSF5 constructs. RT-PCR analysis was performed on proliferating and differentiating cells. In contrast with the clear increase of endogenous mRNA, presented above, this experiment showed that mRNA from mock transfected EGFP construct (not shown) or from EGFP-SRSF5 construct, did not seem to be affected (Figure 3A). Together with data presented above, these results indicate a specific increase of mRNA accumulation from the endogenous SRSF5 gene, and a constant, unregulated expression of transcripts from the transfected cDNA, hence suggesting a promoter-dependent upregulation or post-transcription stabilization of the endogenous SRSF5 gene.

We next examined the protein expression in cells overexpressing a fusion EGFP-SRSF5 protein. Cells overexpressing SRSF5 were cultured in the presence or absence of DMSO for 4 days. Immunoblot analysis using mAb104 revealed a dramatic decrease of EGFP-SRSF5, paralleling that of the endogenous SRSF5 epitopes (Figure S1). Protein analyses were then carried out in a time-course experiment using anti-GFP and anti-SRSF5 antibodies. These experiments reproducibly showed a sharp decrease of protein expression between D2 and D3 of DMSO exposure (Figure 3B). Such decrease was not observed in mock cells, transfected with EGFP construct, nor was it observed in cells overexpressing a fusion EGFP-hnRNPA2 protein (Figure 3B). This pattern was similarly observed in different stable clones, indicating that the fusion protein decrease was not a clone-dependent feature (Figure 3C).

**Figure 4:**
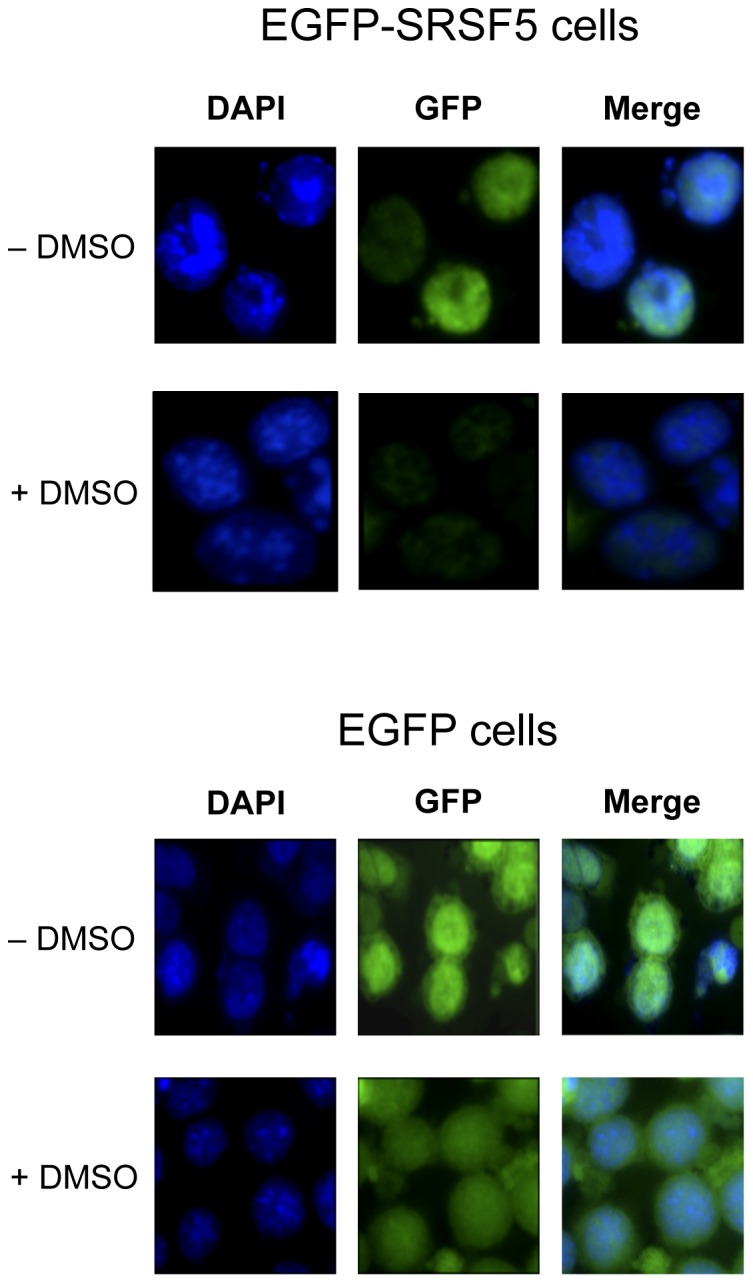
Subcellular localization of SRSF5 during erythroid differentiation. Cells stably expressing EGFP alone (EGFP cells) or the fusion protein EGFP-SRSF5 (EGFP-SRSF5 cells) were stained with DAPI and viewed by fluorescence microscopy. Acquired fluorescence from DAPI-stained nuclei (blue) and emitted from the expression of EGFP (green) shows a strict nuclear localization of SRSF5-containing protein, whereas EGFP protein redistributes to both the nucleus and the cytosol. Note that the fluorescence generated from the fusion protein EGFP-SRSF5 fades away as the cells differentiate, while that emitted from the control EGFP protein remains steady after DMSO exposure.

To examine whether the signal decrease is associated with altered SRSF5 protein distribution in differentiating cells, subcellular expression of fusion EGFP-SRSF5 protein was followed by fluorescent microscopy. The stably expressed protein was consistently expressed in the nucleus with a much fainter staining as the cells differentiated, whereas EGFP alone stained both the nucleus and the cytoplasm, both in uninduced and DMSO-induced cells (Figure 4).

Altogether, these data strongly suggest that SRSF5 protein decreases during erythroid differentiation, and that this decline is tightly dependent on SRSF5 amino acid sequence.

### Post-translation downregulation of SRSF5 is mediated by the proteasome

**Figure 5:**
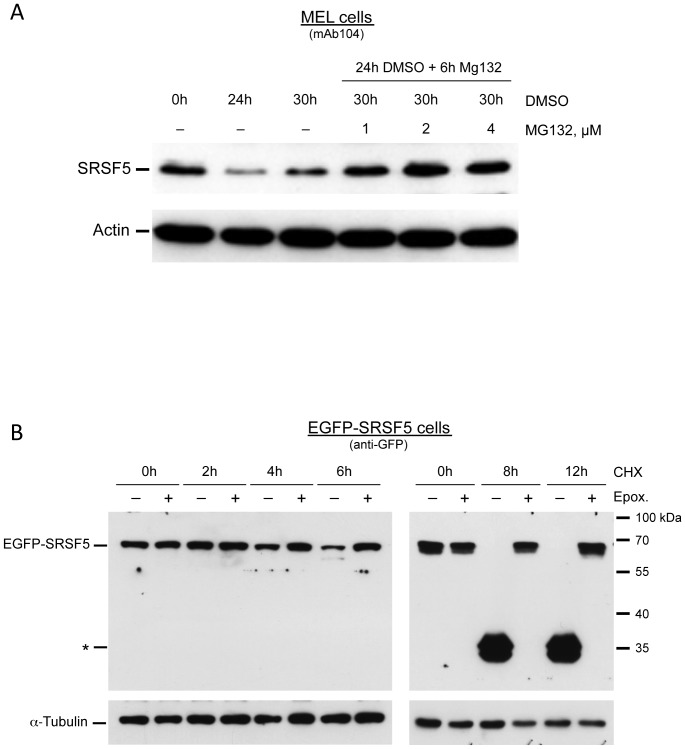
Proteasome-mediated proteolysis of SRSF5 in late erythroid differentiation. A. Proteasome inhibition with MG132. Immunoblot analysis of SRSF5 expression in MEL cells treated with DMSO to induce erythroid differentiation, then with increasing concentrations of MG132 to inhibit degradation by the proteasome. Actin immunoblot was used as a loading control. Note that proteasome inhibition stabilizes SRSF5. B. Cycloheximide chase and proteasome inhibition with epoxomicin in MEL cells. Cells stably transfected with EGFP-SRSF5 construct (EGFP-SRSF5 cells) were induced to erythroid differentiation, and then exposed to cycloheximide (CHX) in a time-course experiment (0 to 12 h exposure). The cycloheximide chase assay showed a decrease of fusion protein, detectable after 4 h of exposure, as revealed by immunoblotting using anti-GFP antibody (− lanes). Degradation fragments (*) appeared at the expense of the full-length protein. This latter completely disappeared 8 h after cycloheximide administration. + lanes correspond to the same cycloheximide experiment performed on cells pre-treated with epoxomicin (Epox.) for 4 h. Cycloheximide was added after epoxomicin removal. In these cells, the fusion protein remained stable over time, providing further support that epoxomicin-mediated inhibition is irreversible, and that SRS5 proteolysis is proteasome-dependent.

Data collected at this stage argue against the possibility that SRSF5 downregulation results from a reduced accumulation of mature mRNA, and stands rather in favor of SRSF5 protein degradation in differentiating cells. To investigate the possible post-translation degradation of SRSF5 by the proteasome, we tested the impact of two different proteasome inhibitors, MG132 and epoxomicin. In MG132 experiments, cells were first induced to erythroid differentiation for 24 h, and then exposed to the proteasome inhibitor MG132. SRSF5 expression was analyzed by immunoblotting experiments. A decrease of SRSF5 signal was detectable within the first 30 h of DMSO induction. Exposure to MG132 resulted in resurgence of SRSF5 to a protein level equivalent to, if not higher than the level observed in proliferating cells (Figure 5A). Moreover, SRSF5 appeared to be stabilized in a MG132 dose-dependent manner. These observations suggest that during late erythropoiesis, SRSF5 is targeted to degradation mediated by the proteasome, causing its sharp post-translation downregulation.

To better monitor SRSF5 degradation over time, we next carried out a cycloheximide chase, wherein cycloheximide was added to cells to block newly translated proteins, and the decay in the steady-state level of the studied protein was analyzed by immunoblotting. In this set of experiments, cells stably transfected with EGFP-SRSF5 construct or the mock EGFP construct were initially pre-treated with DMSO for 34 h to induce erythroid differentiation, and then exposed to cycloheximide for 12 h. The steady-state level of the fusion protein EGFP-SRSF5 was examined by immunoblotting using anti-GFP antibody (Figure 5B, − lanes). It clearly appeared that the fusion protein level decrease occurred after 4 h of exposure to cycloheximide. The full-length fusion EGFP-SRSF5 completely disappeared around 8 h of exposure; concomitantly, degradation EGFP-containing fragments appeared (indicated by a star in Figure 5B). EGFP expressed from the mock cells remained unaltered (not shown), suggesting that proteolytic targeting was dependent on SRSF5 sequence.

A second cycloheximide chase was performed in parallel on cells pre-treated with epoxomicin (Figure 5B, + lanes). Here again, cell differentiation was induced for 30 h with DMSO, prior to exposure to epoxomicin. After 4 h of treatment, the proteasome inhibitor was removed and cells were grown in fresh medium containing both DMSO and cycloheximide, as described above (see also Materials and Methods). Epoxomicin is known to be an irreversible, potent and highly specific inhibitor of the proteasome [26], [27]. The steady-state levels of the EGFP control protein (not shown) and EGFP-SRSF5 fusion (Figure 5B, + lanes) were analyzed by immunoblotting. Contrasting with untreated cells (−lanes), cells exposed to epoxomicin invariably displayed stable and constant level of EGFP-SRSF5 protein.

Collectively, these results are convincing evidence that post-translation downregulation of SRSF5 during erythroid differentiation results from proteasome-mediated proteolysis.

### The RS domain is required for proteasome-mediated proteolysis of SRSF5 in late erythroid differentiation

**Figure 6:**
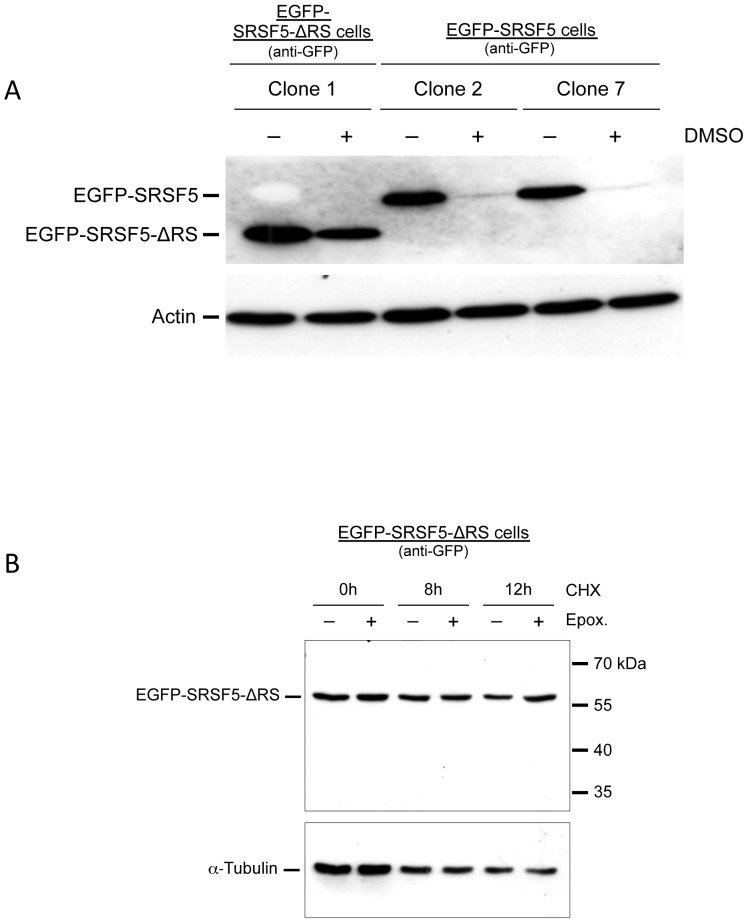
Proteasome-mediated proteolysis of SRSF5 requires the RS domain. A. SRSF5 deprived of the RS domain resists proteasome-mediated degradation. Cells were transfected with the full-length EGFP-SRSF5 fusion or a truncated form missing the RS domain (EGFP-SRSF5-ΔRS). Fusion proteins were analyzed by immunoblotting using anti-GFP antibody. Cells were cultured in the absence (−) or presence (+) of DMSO for 4 days. Actin served to trace loading discrepancies or protein degradation. B. Cycloheximide chase and proteasome inhibition with epoxomicin. The experiment was performed on EGFP-SRSF5-ΔRS cells, as indicated in Materials and Methods and in Figure 5 legend. Data are to be compared with cycloheximide chase and proteasome inhibition experiments on EGFP-SRSF5 cells, shown in Figure 5B. Note that RS domain-lacking proteins in EGFP-SRSF5-ΔRS cells are not intercepted by the proteasome-induced proteolysis (see also Text). Antibodies are indicated between parentheses.

The SR proteins are characterized by a C-terminal region enriched in Arg-Ser dipeptides (RS domain) [6]. We tested the expression status of SRSF5 deprived of its RS domain in cells induced to erythroid differentiation. Cells were stably transfected with either the cDNA encoding the full-length SRSF5 fused to EGFP (EGFP-SRSF5), or a cDNA encoding a truncated form missing the RS domain (EGFP-SRSF5-ΔRS). Western blot analysis using an anti-GFP antibody revealed specifically the expression of the fused proteins in cells cultured in proliferation medium condition (Figure 6A). Induction of the cells to erythroid differentiation resulted again in a sharp decrease in the expression of the full-length fusion EGFP-SRSF5, whereas the EGFP-SRSF5-ΔRS fusion protein remained rather stable, despite a slight decrease was detectable after 4 days of DMSO-induction of the cells (Figure 6A).

We next analyzed the accumulation of EGFP-SRSF5-ΔRS fusion protein in a cycloheximide chase assay as described above. The experiment was restricted to 3 points: 0, 8 and 12 h, following cycloheximide administration. As shown in Figure 6B, −lanes, the fusion protein missing the RS domain remained more stable and no degradation products emerged, in comparison with the complete vanishing of the full-length protein (Figure 5B). As expected, pre-treatment with epoxomicin did not change the pattern of EGFP-SRSF5-ΔRS protein accumulation (Figure 6B).

Collectively, these observations suggest that SRSF5 proteolytic downregulation is mediated by the proteasome during late erythropoiesis, and that this post-translation downregulation necessarily entails an intact RS domain of the protein.

### The CLK and AKT phosphorylation pathways are not required for proteasome-mediated proteolysis of SRSF5 in late erythroid differentiation

**Figure 7:**
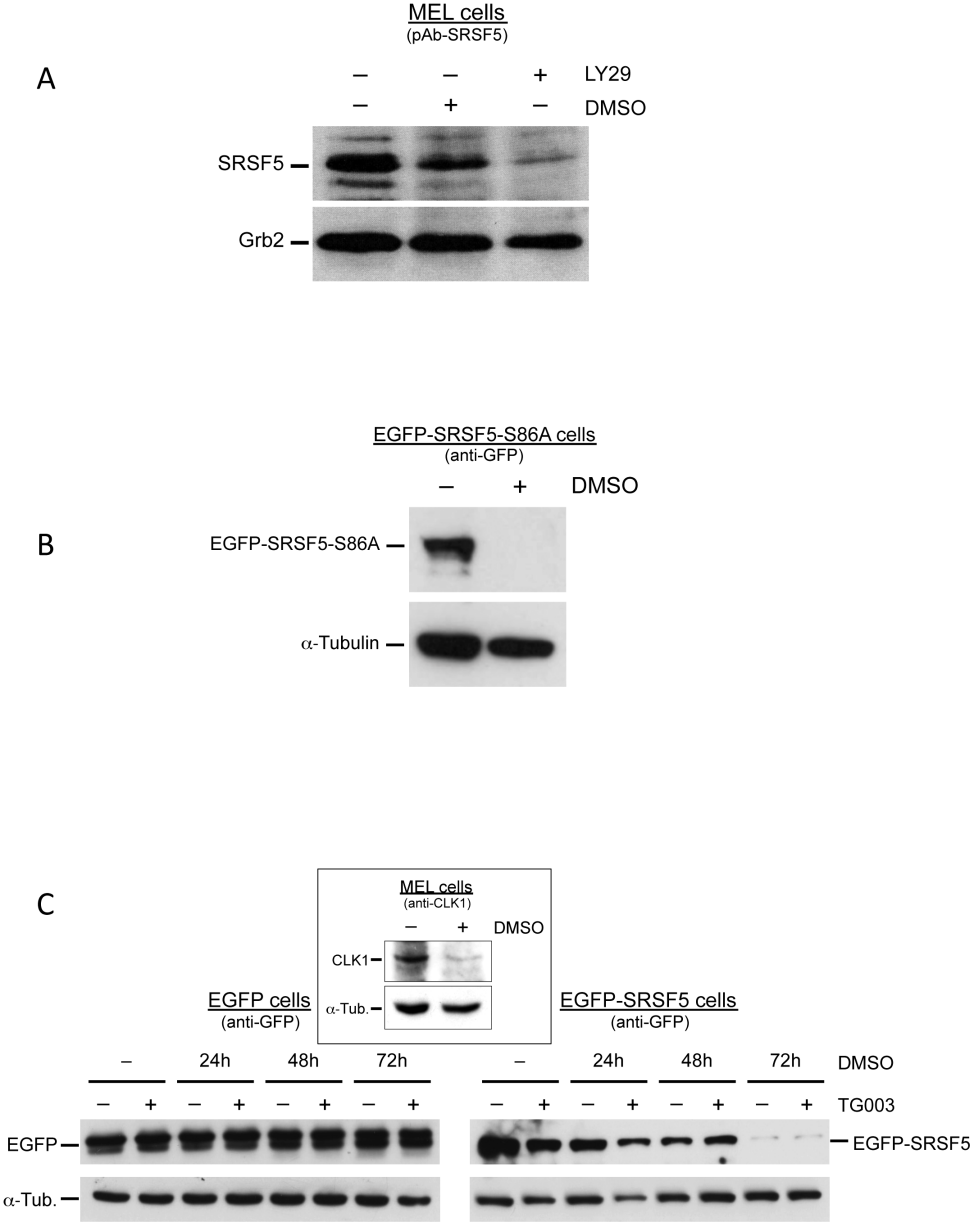
Phosphorylation by the CLKs or by AKT is not required for proteasome-mediated degradation of SRSF5. A. Inhibition of PI3K/AKT signaling. MEL cells were treated for 4 days with either DMSO or LY294002 (LY29), a PI3K/AKT inhibitor. Immunoblot analysis of SRSF5 expression was revealed by anti-SRSF5 antibody (pAb-SRSF5). The decrease in SRSF5 accumulation is most likely secondary to cell differentiation triggered by PI3K/AKT inhibition [31]. Grb2 immunoblot served as control. B. Mutation of AKT phosphorylation site Ser86. Cells stably expressing the recombinant SRSF5 protein mutated at position 86 (EGFP-SRSF5-S86A cells) were treated with DMSO for 4 days, and the fusion protein assessed by immunoblot analysis using anti-GFP antibody. Alpha-tubulin immunoblot was used as control. Abolished AKT phosphorylation site at Ser86 did not affect the regulated post-translation downregulation of SRSF5. C. Inhibition of CLKs. Cells expressing either EGFP alone or the fusion protein EGFP-SRSF5 were analyzed by immunoblotting using anti-GFP, in a time-course DMSO induction experiment (0 to 72 h of exposure), combined with a 6 h exposure of TG003, a CLK inhibitor. Absence (−) or presence (+) of the inhibitor are indicated. Downregulation of SRSF5-containing protein correlated with exposure to DMSO, rather than with TG003 treatment. Expression of CLK1 was assessed in MEL cells untreated (−) or treated for 4 days (+) with DMSO, using anti-CLK1 antibody (insert).

Having established the importance of the RS domain in proteasome targeting of SRSF5 to degradation, we asked whether this feature implies the phosphorylation state of SRSF5. The extensive serine phosphorylation of the RS domain is important in regulating the activities and localization of SRSFs (see Introduction; reviewed in [2], [12], [14]). It has been documented that SRSF5 is a phosphorylation target of AKT, the downstream effector of PI3K [28]–[30]. We analyzed SRSF5 expression using an anti-SRSF5 specific antibody in cells cultured in the presence of LY294002, a commonly used inhibitor of PI3K/AKT signaling cascade. Immunoblotting experiments revealed a clear decrease in SRSF5 epitopes (Figure 7A). knowing that inhibition of the PI3K/AKT signaling cascade triggers MEL cell erythroid differentiation [31], it would be difficult to conclude whether SRSF5 downregulation is a direct effect of a phosphorylation suppression due to PI3K/AKT inhibition, or an indirect effect of induced cell differentiation.

SR proteins contain multiple AKT phosphorylation consensus RXRXX(S/T) sequences. Among these phosphorylation motifs, it has been shown that AKT phosphorylates specifically the Ser residue 86 in the RS domain of SRSF5. Mutation of Ser86 to Ala abolishes AKT phosphorylation of SRSF5 [30]. A new construct, named EGFP-SRSF5-S86A, was obtained by directed mutagenesis as to contain an Ala at position 86 instead of Ser. MEL cells stably transfected with this construct were cultured in the presence or absence of DMSO. Expression of the fusion protein was assessed by immunoblotting using anti-GFP antibody. This experiment revealed a complete disappearance of the mutated fusion protein in cells DMSO-induced to differentiation for 4 days (Figure 7B), indicating that abolished phosphorylation site at Ser86 does not modify the fate of SRSF5 in differentiating cells.

Among the SR protein-specific kinases, the CDC2-like kinases (CLKs) are key factors that enable SR proteins to control pre-mRNA splicing in response to phosphorylation, predominately on serine residues (see [32] and references therein). CLKs themselves are phosphorylated on serine/threonine and tyrosine residues [33]. To distinguish between a direct phosphorylation of SRSF5 by PI3K/AKT and an indirect effect through PI3K/AKT-mediated activation of CLK, the CLK inhibitor TG003 was used. TG003 is a potent inhibitor of all CLK family members, except CLK3 [34]. First, we sought to apprehend the expression of CLK in differentiating MEL cells. As shown in Figure 7C, immunoblot analysis using anti-CLK1 antibody revealed a dramatic decrease in CLK1 expression during late erythroid differentiation, suggesting that CLK1-mediated phosphorylation might not be critical for proteasome-induced proteolysis of SRSF5.

Expression pattern of the fusion protein EGFP-SRSF5 was analyzed by immunoblotting in a time-course DMSO-induction of erythroid differentiation, in the presence or absence of 10 µM TG003 inhibitor. The drug was added to culture medium 6 h before harvesting the cells. Expression of exogenous EGFP protein in mock cells was invariably constant and independent of the presence or absence of TG003, whereas EGFP-SRSF5 cells displayed a decrease of the fusion protein. This decrease was rather correlated with the time of exposure to DMSO, rather than with the presence or absence of the CLK inhibitor TG003 (Figure 7C). This experiment further supports the view that phosphorylation by the CLKs is not required for proteasome-induced proteolysis of SRSF5 in late erythroid differentiation.

### SRSF5 is a potential activator of 4.1R exon 16 splicing in proliferating erythroid cells

To investigate the impact of SRSF5 regulated expression on pre-mRNA splicing in erythroid cells, we undertook a functional analysis on alternative splicing of the endogenous protein 4.1R exon 16. This splicing event is the best characterized in the erythroid system: exon 16 is skipped in early progenitors and is massively retained in mature erythroblasts, which enables the synthesis of a protein isoform with a functional 10 kDa internal domain, needed to stabilize the spectrin-actin complex of the membrane skeleton ([35], [36], and references therein). MEL cells can reproduce this feature upon induction to terminal differentiation using DMSO, either from the endogenous gene [35] or from a transfected minigene [36].

**Figure 8:**
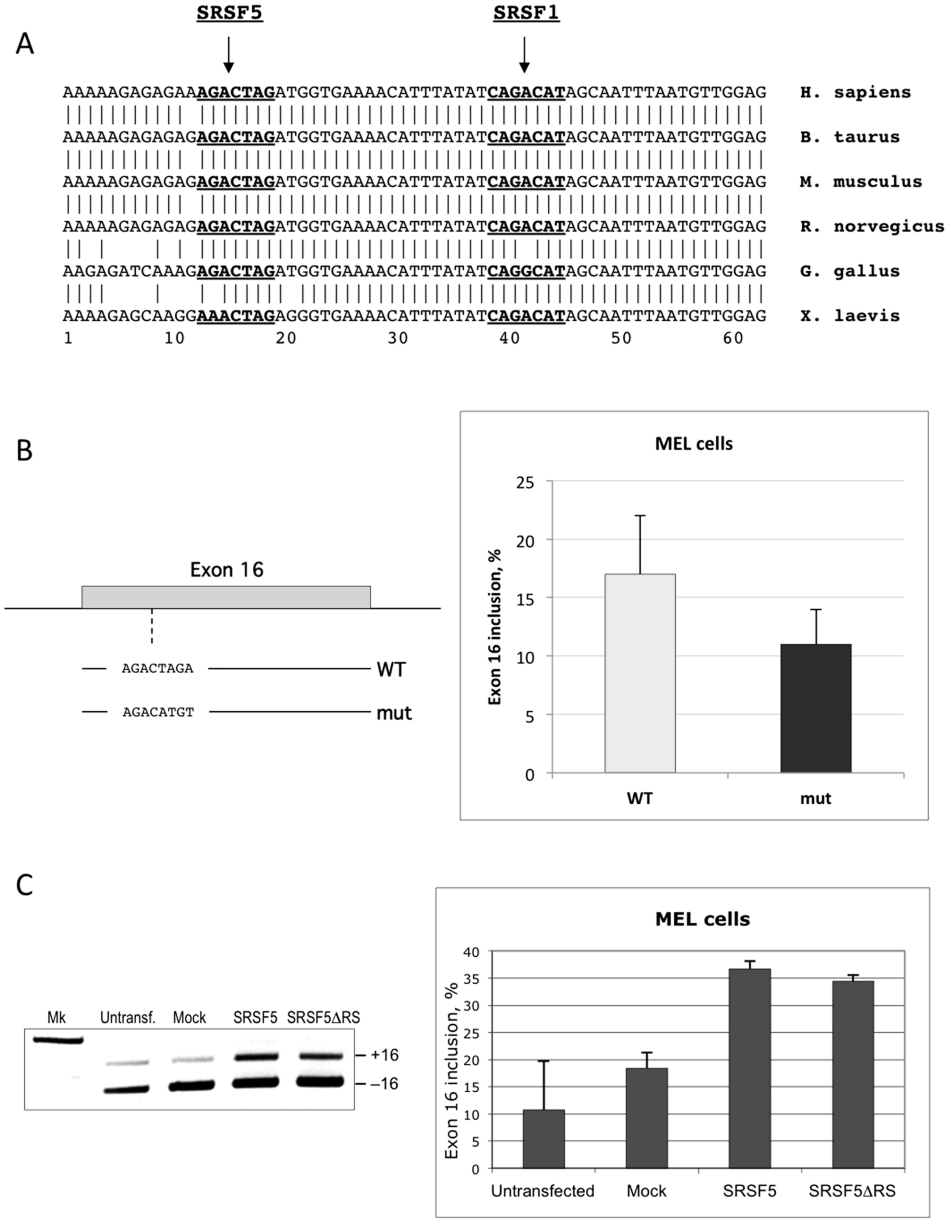
Functional analysis of SRSF5 on endogenous pre-mRNA splicing. To examine the impact of SRSF5 expression on splicing, we used 4.1R exon 16 as a model for endogenous erythroid splicing event.A. 4.1R exon 16 sequence in vertebrates. Nucleotide sequence alignment in vertebrates displays a very conserved exon 16 sequence. ESEfinder revealed two distinct conserved motifs (underlined bold sequences), potential recognition sites for 2 members of the SR family: SRSF5 and SRSF1 (see also [37]). B. Impact of ESE alteration on exon 16 splicing. The motif identified as SRSF5 ESE was altered by targeted mutagenesis. The mutated exon 16 and its flanking intronic sequences were inserted in a splicing cassette [36], the resulting mutated minigene (mut) was stably transfected in MEL cells, and exon 16 splicing was analyzed. Data were compared to unaltered exon 16 splicing from wildtype minigene construct (WT). C. Exon 16 splicing pattern in cells overexpressing the full-length protein or the RRM domains of SRSF5. Exon 16 splicing was analyzed in single tests (left panel) or in semi-quantitative experiments (right panel) in cells overexpressing either the full-length SRSF5 (SRSF5) or a shorter form missing the RS domain SRSF5 (SRSF5ΔRS). Data are to be compared with untransfected cells or cells overexpressing EGFP only (Mock).

A previous study has shown that SRSF1 binds the ESE sequence CAGACAT within exon 16, and promotes exon inclusion in vitro and in intact cells [37]. In addition to the SRSF1 binding site, exon 16 exhibits another motif that can potentially function as an ESE through binding to SRSF5, as suggested by bioinformatics sequence analysis using ESEfinder software (Figure 8A; [37]). This site also appears to be conserved among vertebrate species. In a first set of experiments, we altered the potential binding site for SRSF5, AGACTAG, by directed mutagenesis using a minigene template that reproduces the regulated splicing switch of exon 16 in stably transfected MEL cells [36]. Exon 16 was analyzed by calculating the percentage of splicing inclusion (Ψ, [38]). As shown in Figure 8B, disruption of the SRSF5 binding site resulted in reduced exon 16 inclusion. These data imply that SRSF5 may have a potential activating effect on exon 16 splicing.

To further test the effect of SRSF5 on exon 16 splicing, inclusion of the exon was monitored in cells stably transfected with recombinant plasmid expressing SRSF5. As shown in Figure 8C, overexpression of SRSF5 clearly activated exon 16 inclusion in proliferating MEL cells. Interestingly, deletion of the RS domain of SRSF5 spares the N-terminal RNA-binding domains RRM1 and RRM2, which potentially recognize the AGACTAG motif in exon 16. As shown both in single tests and in semi-quantitative analysis (Figure 8C), overexpressing an SRSF5 lacking the RS domain activated exon 16 inclusion in MEL cells, with almost the same efficiency as the full-length protein (see Discussion).

Since SRSF5 is basically expressed only in pre-differentiated cells, we finally examined the impact of SRSF5 knockdown on exon 16 splicing in these cells. SiRNA targeting SRSF5 (siSRSF5, Materials and Methods) were transfected in MEL cells overexpressing EGFP-SRSF5 fusion protein. SRSF5 mRNA and protein levels of expression were assessed 24 h and 48 h after transfection. As evidenced by real-time RT-PCR, SRSF5 mRNA dramatically decreased within the first 24 h after siSRSF5 transfection, and remained low within the next 24 h (Figure S2A). Concomitant decrease of both the endogenous and the exogenous SRSF5 proteins was ascertained by immunoblotting experiments (Figure S2B). 4.1R exon 16 inclusion was estimated in siSRSF5 transfected cells by semi-quantitative RT-PCR (Figure S2C). Expectedly, this experiment did not show a significant change in the percentage of exon inclusion. In fact, it was difficult to clearly show that SRSF5 knockdown further lowers an otherwise low percentage (0–5%) of exon 16 containing isoforms.

Altogether, these data further support that SRSF5, or its RNA-binding domains, have the potential to activate the endogenous exon 16 splicing in proliferating erythroid cells.

### SRSF5 is not required for exon 16 splicing in differentiating erythroid cells

**Figure 9:**
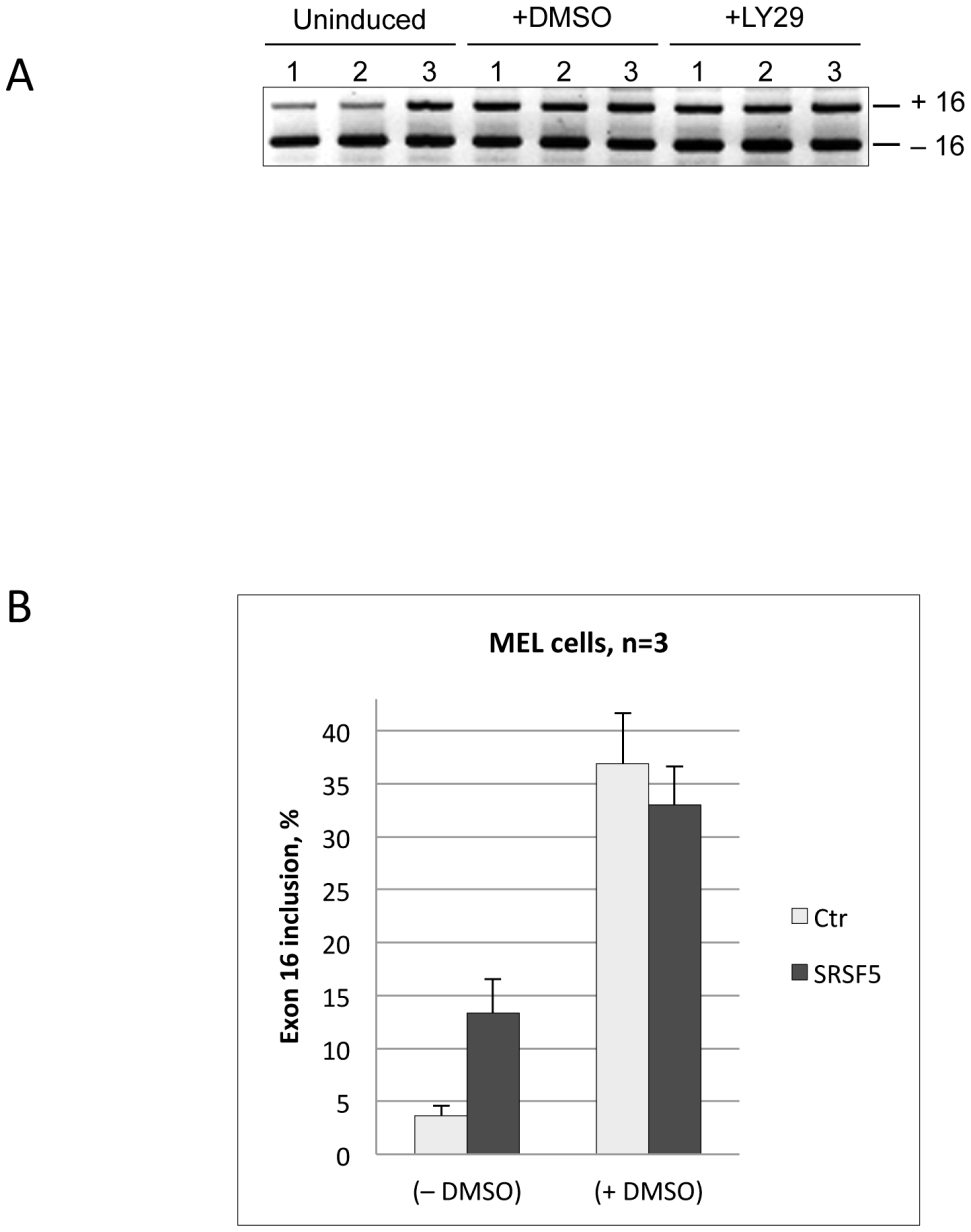
Impact of SRSF5 overexpression on endogenous pre-mRNA splicing during erythroid differentiation. A. Electrophoretic analysis of exon 16 splicing in cells overexpressing SRSF5. Cells were cultured either in the absence (uninduced) or presence of DMSO (+DMSO) or PI3K inhibitor LY294002 (+LY29) for 4 days. Both agents trigger cell erythroid differentiation [31]. Exon 16 splicing was analyzed in untransfected cells (1), cells transfected with mock vector containing only EGFP (2), and in cells transfected with a recombinant vector overexpressing the fusion protein EGFP-SRSF5 (3). B. Semi-quantitative analysis of exon 16 splicing in cells overexpressing SRSF5 before (-DMSO) or after (+DMSO) DMSO-treatment. Exon inclusion is to be appraised comparing to control Mock cells (Ctr).

Previous studies have shown that the regulated splicing of exon 16 in late erythroid differentiation involves at least 2 activating factors: SRSF1 and Fox2, which recognize enhancer sequences within the exon (Figure 8A) and the downstream intron, respectively [37], [39]–[41]. Concomitance of proteasome-mediated proteolysis of SRSF5 and exon inclusion during late erythroid differentiation, rationally suggests that SRSF5 must have no effect on exon inclusion in differentiating cells. To test this hypothesis, exon 16 splicing was examined in untransfected MEL cells or cells transfected with either the mock EGFP construct or EGFP-SRSF5 construct. Exon splicing was analyzed before and after DMSO treatment (Figure 9). Again, exon inclusion was clearly enhanced in proliferating cells. As expected, no effect was noticeable in cells overexpressing SRSF5, in comparison with untransfected cells or cells transfected with the mock construct.

As mentioned above, extensive studies have documented that SRSFs are active in the phosphorylated form, and their phosphorylation is crucial for RNA-binding specificity, splicing activity and for their subcellular localization (reviewed in [2], [12], [14]). In particular, phosphorylation of SRSF5 in response to the PI3K signaling pathway has been shown to be associated with a change in alternative splicing [28]–[30]. Our recent data have also demonstrated that inhibition of the PI3K/AKT pathway using LY294002 is sufficient to activate the erythroid splicing of 4.1R exon 16 [31]. We therefore tested the impact of LY294002-mediated inhibition of the PI3K/AKT signaling cascade in cells overexpressing SRSF5. In agreement with the data gathered above, this experiment showed a splicing pattern of exon 16 similar to that observed in DMSO-induced cells (Figure 9A).

In conclusion, consistently with the downregulation of SRSF5 during erythroid differentiation, this SR protein appears from our experiments to have a marginal, if any, impact on the erythroid switch in exon 16 splicing.

## Discussion

Numerous studies have concurred to the idea that abundance and post-translation modifications, mainly phosphorylation, are the major factors that modulate SR protein activity [2], [5], [12]. In fact, modulation of SR protein levels has started being considered as a parameter for characterizing human pathologies. Thus, increased expression of several classical SR proteins has been observed, in correlation with cancer progression in several human tumors, including ovary, lung, colon, kidney, liver, pancreas and breast cancer; however, the mRNA levels of SRSF1, SRSF5, SRSF6 and SRSF4 are lower in non-familial colon adenocarcinomas than in healthy tissue (reviewed in [2], [5], [42]).

While extensive studies have documented that reversible phosphorylation is essential for functional SR proteins [12], only few studies have addressed the post-transcription regulation of SR protein levels of expression. This emerging theme is now attracting interest, and data started to accumulate. In fact, different mechanisms can modulate the cell levels of functional SR proteins: (i) A specific promoter can modulate SR protein expression. It has been shown that the transcription factor E2F1 upregulates *SRSF2* gene [43]. Similarly, the HPV transcription factor E2 binds and transactivates a subset of *SRSF* genes, including *SRSF1*, *SRSF2* and *SRSF3*, in infected epithelial cells [44]. (ii) Autoregulatory negative mechanisms, mainly through alternative splicing-coupled NMD, decrease SR protein expression [45]–[47]. SRSF2, for instance, negatively controls its own expression, through activation of alternative splicing events leading to the generation of distinct nonsense mRNA isoforms that are targeted by NMD [45]. (iii) A non-coding RNA can interfere with full-level expression of an SR protein. An abundant mammalian RNA, metastasis-associated lung adenocarcinoma transcript 1 (MALAT1), predominantly localized to nuclear speckles, interacts with SRSF1, SRSF2, and SRSF3, but not SRSF5, and modulates their distribution to nuclear speckles. MALAT1 further regulates endogenous pre-mRNA alternative splicing by controlling the functional levels of phosphorylated SR splicing factors [48]. (iv) A proteasome-related mechanism can decrease SR protein expression or perturb their function. Hence, proteasome-PA28γ complexes regulate nuclear speckle organization, and therefore govern intranuclear trafficking of SR proteins [49]. More recently, the acetyltransferase Tip60 was found to acetylate SRSF2, and promote its proteasomal degradation [50].

Proteasomal degradation has been described as a post-translation, yet irreversible, mechanism that regulates numerous transcription factors, and consequently modulates the expression of their target genes (see [51] for review). However little is known regarding a proteasomal regulation of splicing factors, such as the SR protein family. Aside from their function as splicing factors, the SR proteins are emerging as master regulators of gene expression [5], [9], [10]. In this study, we explored the post-translation regulation of SRSF5 expression in the context of late erythroid differentiation. We documented that the endogenous and the transfected SRSF5, both were targeted to proteolytic downregulation as the cells underwent terminal differentiation. Use of one of the most selective proteasome inhibitors demonstrated that SRSF5 proteolysis was indeed dependent on proteasome activity. We provided compelling evidence that this feature was not an epiphenomenon occurring in chemically-induced, EPO-independent erythroleukemia cells, but a real cellular event that accompanies the late differentiation of MEL cells, and that of EPO-dependent primary erythroblasts as well. Henceforth, SRSF5 shut-off event will provide another marker of late erythroid development. Cells blocked in their differentiation, such as MEL cells, would on the contrary maintain a high level of SRSF5.

The proteasome is the pivotal component in cytosolic protein degradation. Malfunction of the ubiquitin proteasome system is associated with various disease conditions, including hematopoietic malignancies (for review, see [52]). We are only beginning to appreciate the importance of this post-translation regulation system in erythroid development. Only a handful of studies have addressed the proteasomal regulation in erythroid cells. It has been documented, for instance, that interregulated protein quality control leads to degradation of excess α-globin in β-thalassemia [53]. Another recent study has reported that a phosphorylated form of HSP27 generated in a p38-dependent manner binds to acetylated GATA-1, in late stages of erythroid differentiation, to promote its ubiquitination and proteasomal degradation [54]. Lee et al. have provided evidence that turnover of the p45 protein in undifferentiated MEL cells is regulated through the ubiquitin proteasome system in a phosphorylation-dependent manner: p54 is phosphorylated by the P-JNK. During MEL cell differentiation, P-JNK is inactivated, and this leads to p45 stabilization during the early phase of differentiation [55].

Although SR proteins are ubiquitously expressed, they were reported to exhibit differential expression in certain tissues and cell types in response to signaling [2], [13]. From our data, it appears that the SR protein phosphorylation activity mediated by PI3K/AKT signaling and CLK1 decreases during erythroid differentiation. We hypothesized that modulation of the phosphorylation might impact the stability of SRSF5. We found that, in fact, the presence of the RS domain was critical for SRSF5 downregulation. However, neither phosphorylation of SRSF5 by the CLKs, nor phosphorylation by AKT of the major site Ser86, affected SRSF5 stability.

In an attempt to explore the impact of the post-translation proteolysis of SRSF5 on splicing, we investigated the changes in alternative splicing of endogenous pre-mRNA. SRSF5 appeared as a potential activator of 4.1R exon 16. Alteration of a favorable SRSF5 binding sequence resulted in exon splicing inhibition. Consistently, overexpression of SRSF5 enhanced wildtype exon inclusion in proliferating cells.

The ability of SR proteins to bind a pre-mRNA is essential for their activity in both constitutive and alternative splicing ([56]–[58]; for review, see [2]). The RRMs of SR proteins mediate sequence-specific binding to the RNA, and thereby determine the substrate specificity, whereas the RS domains participate in protein-protein interactions [59], [60]. Moreover, the RS domain seems to be important for constitutive splicing, but dispensable in alternative splicing [56], [61]. Interestingly, we found that overexpression of SRSF5 missing the RS domain efficiently activates endogenous pre-mRNA splicing. This observation is in keeping with previous studies showing that SR proteins lacking the RS domain may be sufficient to compete with the binding of antagonistic splicing factors to adjacent splicing silencer sequences [8], [62]. This is in further agreement with the observation that an SRSF1 mutant lacking the RS domain could rescue cell viability in SRSF1-depleted mouse embryo fibroblasts [63], suggesting that defects in alternative splicing are incompatible with proper cellular functioning. Equally possible is the fact that a defective interaction of SRSF5ΔRS with other factors involved in exon 16 splicing regulation, may preclude efficient exon inclusion.

In the erythroid system, we have documented that constitutive PI3K/AKT sustains Spi-1/PU.1 autoregulated expression that inhibits protein 4.1R exon 16 splicing and erythroid differentiation, and that inhibition of PI3K/AKT signaling blocks Spi-1/PU.1 autoregulation loop, and triggers 4.1R erythroid splicing activation [31]. Overexpressed SRSF5 most likely undergoes the same proteasome-mediated degradation pathway in DMSO-induced cells, which abrogates any additional effect on exon 16 after cell induction. The decrease was observed with either an antibody against the phosphorylated forms or an anti-GFP antibody, suggesting that the SRSF5 downregulation is not due to a change in the phosphorylation status of the protein. Subcellular localization of the EGFP-SRSF5 fusion protein further argued in favor of a protein quantitative downregulation and against a dephosphorylation-mediated regulation. This observation further suggest that, beyond their role in pre-mRNA splicing regulation, the protein-protein interaction properties of the RS domains should now be extended to their role in targeting a subset of SR proteins to proteasome-induced proteolysis.

The biological significance of the mRNA upregulation remains to be elucidated. The opposite pattern of expression, observed here between SRSF5 mature transcripts and proteins seems puzzling. However, earlier concepts of mRNA transcripts exclusively as a support of protein synthesis code have evolved to a new view of mRNA as a multifunctional molecule [64]. Further investigations of this hypothesis are to be conducted regarding SRSF5 mRNA in the erythroid system. Nevertheless, the opposite pattern of expression of SRSF5 mRNA and protein observed here, questions the relevance of transcriptomic global studies with no further analysis of protein expression and function.

Further investigations are warranted to address the role of SRSF5 in normal erythroid and leukemic proliferation, and the impact of a functional form of SRSF5, yet safeguarded from proteasome targeting, on cell differentiation, and to precisely identify the proteasome target sequence within the RS domain of SRSF5.

## Materials and Methods

### Ethics statement

This study was approved by the Université Lyon 1 and the Centre National de la Recherche Scientifique (CNRS). The investigations were conducted in accordance with the French legislation, and the "National Charter" of the "French National Committee for Consideration of Ethics in Animal Experimentation", edited by the Ministère de l'Enseignement Supérieur et de la Recherche, Direction de la Recherche et de l'Innovation", and the "Ministère de l'Agriculture et de la Pêche" (Licence # 4936, related to handling animals, cell lines, viruses, microorganisms and recombinant DNAs).

Mouse eythroleukemia cell line generation has been previously described [23], and widely utilized afterwards by different groups, including ours [31], [65], [66].

### Plasmid constructs

A construct previously described [36] will be referred to as wild-type (WT) minigene. It was obtained by inserting a 0.7 kb PCR-generated genomic fragment containing protein 4.1R exon 16 at the *Bst*EII/*Nhe*I site of the splicing cassette p(13,17)/CMV. A mutant construct (mut) was also prepared as *Bst*EII/*Nhe*I fragment and subcloned in p(13,17)/CMV. The mutation was generated by a PCR site-directed mutagenesis procedure using complementary mutant primers F1 and R1 (Table S1), and the WT construct as template (QuickChange Site-Directed Mutagenesis Kit, Agilent Technologies-*Stratagene* Products division, Waldbronn, Germany). The WT and the mutant inserts were fully sequenced to ascertain the absence of any additional mismatch.

Recombinant plasmids expressing enhanced green fluorescent protein (EGFP) fused to either the full-length SRSF5 or a shorter form missing the RS domain, were obtained as follows: The entire coding sequence of SRSF5 was amplified by RT-PCR using primers F2 and R2 (Table S1). A fragment lacking the RS domain-encoding sequence was generated by RT-PCR using primers F2 and R3 (Table S1). *Bsp*EI and *Eco*RI restriction sites were added at the 5′ ends of the forward and reverse primers, respectively, to ease fragment cloning. The PCR products were inserted at the *Bsp*EI/*Eco*RI site of PEFbosEGFP-C1 expression vector [31], in continuous open reading frame with the EGFP. The resulting constructs will be called EGFP-SRSF5 and EGFP-SRSF5-ΔRS, respectively. An additional construct was prepared to serve as a control; it contained the coding sequence for hnRNPA2 splicing factor, fused to EGFP (A. Douablin, Ph.D. Thesis).

EGFP-SRSF5-S86A construct was generated by 2 step-PCR site directed mutagenesis, using complementary mutant primers S86A-S and S86A-AS (Table S1), MEL cell total RNA as template and KAPA HiFi Hot Start Ready Mix (KAPA Biosystems, Woburn, MA). The first PCR step used EGFP-SRSF5 construct as template and forward and reverse primers F2 and S86A-AS on one hand and S86A-S and R2 on the other hand. The resulting fragments were cut out of agarose gel and subsequently used as templates in a second step PCR using primers F2 and R2 (Table S1). The generated PCR product was digested by *Bsp*EI and *Eco*RI and subcloned in PEFbosEGFP-C1 vector. The insert was entirely sequenced to ascertain the presence of the single base substitution responsible of the Ser→Ala amino acid change at position 86, and the absence of any other nucleotide sequence changes.

### MEL cell culture and induction of erythroid differentiation

Mouse erythroleukemia (MEL) cell subclone 745A used in this study is able to undergo terminal differentiation upon chemical induction [66]. Cells were cultured and induced to erythroid differentiation in the presence of DMSO or LY294002, a specific inhibitor of the PI3K/AKT signaling, as previously described [31], [65]. Cells were collected for protein and mRNA analyses at day zero and after 2–4 days of induction.

### Cell transfection and selection of stable clones

For overexpression experiments, MEL cells were stably transfected with EGFP-SRSF5, EGFP-SRSF5-ΔRS, EGFP-hnRNPA2, EGFP-SRSF5-S86A, or EGFP mock construct, using ESCORT (Sigma) or DreamFect Gold (OZ Biosciences, Marseille, France) reagents. The transfection and clone selection procedures were as previously described [31]. Instant immunofluorescence microscopy observations helped to appraise the expression of the exogenous fusion proteins and the transfection efficiency. Expression of the fusion proteins and recombinant mRNAs was subsequently analyzed by immunoblotting and RT-PCR approaches.

### NMD inhibition

To block the NMD mechanism, cells were treated in culture with the 2 drugs cycloheximide and caffeine, as previously indicated [24].

### Proteasome inhibition *in cellulo*

MEL cells were treated with a reversible proteasome inhibitor, MG132 (Sigma) or a highly selective and irreversible inhibitor, epoxomicin (Sigma). Cells were cultured for 24 h in the presence of DMSO to trigger erythroid terminal differentiation, and then treated for 6 h with 1, 2, or 4 µM final concentration of MG132, in the same differentiation culture medium. Cells were harvested by centrifugation and the pellets were washed twice with 1×PBS, and lysed for protein extraction and analysis. For epoxomicin inhibition, the experiments were carried out on MEL cells stably transfected with EGFP mock, EGFP-SRSF5, or EGFP-SRSF5-ΔRS constructs. Cells (1×10^6^) were seeded on 6 well plates and grown for 30 h in differentiation complete medium (DMEM, 10% FBS, containing 1.8% DMSO and 500 µg/ml G-418). Epoxomicin was added at 2 µM final concentration, and cells were further incubated for 4 h, and then washed twice with 1×PBS. Cell pellets were resuspended in differentiation culture medium, supplemented with cycloheximide (Sigma) at 30 µg/ml final concentration, and incubated for 2 to 12 h. Cells were harvested at different time points, washed twice with 1×PBS, and the pellets quick-frozen in liquid nitrogen, and stored at −80°C for subsequent protein extraction and analysis. Treatment with cycloheximide was carried out in parallel on cells that were not exposed to epoxomicin. This experiment served as a cycloheximide chase to follow the degradation of the steady-state protein.

### Inhibition of CLK in cultured MEL cells

MEL cells stably transfected with EGFP mock construct or the EGFP-SRSF5 construct were seeded in 12 well plates (2×10^5^ cells/well), and grown in the absence or presence of 1.8% DMSO to induce erythroid differentiation. SR protein kinase inhibitor TG003 was added at 10 µM final concentration 6 h before harvesting the cells. A time-course DMSO-induction experiment was carried out on cells in the presence or absence of TG003. Cells were collected, extensively washed with 1×PBS, and the pellets were quick-frozen for subsequent analyses.

### Ex-vivo primary erythroblast proliferation, maturation and treatment

Extensively self-renewing erythroblasts (ESREs) were derived from E13.5 mouse embryonic fetal liver, as previously described [25], using STEMPRO34 medium. These cells are dependent on erythropoietin (EPO), stem cell factor (SCF), and dexamethasone for their ex-vivo self-renewal. Proliferating erythroblasts mature into erythrocytes when cultured in the absence of dexamethasone, in erythroid maturation media, which contains IMDM, EPO, SCF, serum replacement, PDS (plasma-derived serum), glutamine, PFHM-II (protein-free hybridoma media) and MTG (monothioglycerol). Detailed protocol has recently been described [25]. Cell size was measured using forward scatter parameter (FSC). ESRE cell differentiation was monitored using a FACSCanto II flow cytometer (BD Biosciences, Le Pont-De-Claix, France) after triple labeling with the following monoclonal conjugated antibodies: anti-CD71–fluorescein isothiocyanate (FITC), anti-Ter119–phycoerythrin (PE) and anti-CD117-allophycocyanine (APC). All these antibodies were purchased from Caltag Laboratories (Burlingame, CA). Cells were collected for protein and RNA analyses.

### siRNA-mediated knockdown

The complementary SRSF5-specific siRNAs (5′-CCUCGAAAUGAUAGACGAA-3′) and (5′-UUCGUCUAUCAUUUCGAGG-3′), along with irrelevent siRNA (5′-GCAAGCUGACCCUGAAGUUCAT-3′) were designed by, and purchased from Eurogentec (Eurogentec S.A., Seraing, Belgium). Exponentially growing cells were concentrated to 10^7^ cells/mL in culture medium, and 500 µl of cell suspension was pipetted into a 4-mm electroporation cuvette (Ozyme/Clonetech). Immediately before the electroporation step, siRNAs were added, at a final concentration of 500 nM. Electroporation was performed with BTX ECM 830 Square Electro Porator (BTX, Harvard Apparatus) using a rectangle pulse of 300 V (pulse length 10 ms). After incubating for 15 minutes at room temperature, the cells were diluted 20-fold with culture medium and incubated at 37°C and 5% CO2. Cells were collected 24 h and 48 h after siRNA administration, for RNA and protein analyses.

### mRNA isolation and analysis

Total RNA was isolated from cultured MEL cells, or fetal liver erythroid progenitors using the TRIzol Reagent (Life Technologies SAS, Villebon-sur-Yvette, France). RT-PCR experiments were performed for qualitative purposes to analyze SRSF5, SRSF3, and actin expression. The protocols were basically as previously described [31]. Appropriate forward and reverse primers F4/R4, F5/R5, and F6/R6 (Table S1) were used to amplify SRSF5, SRSF3 and β-actin cDNA fragments, respectively.

Semi-quantitative RT-PCR served to assess the percentage of exon 16 inclusion as a ratio of exon 16-containing isoform to total 4.1R isoforms. This parameter has been recently termed the percent splicing index, Ψ [38]. The experimental protocol was as previously described [36]. PCR primers were chosen as to amplify specifically either the endogenous- or the minigene-derived transcripts [36].

Real-time RT-PCR was used to quantify SRSF5 and SRSF3 mRNA accumulation during erythroid development. The experimental protocol was as recently detailed [31]. Forward and reverse primer sets F7/R7, F9/R9 and F6/R6 were used to amplify SRSF5, SRSF3 and β-actin mRNAs, respectively (Table S1). Actin served as an internal control.

### Protein analysis

Proteins were extracted from cultured cells as follows: cells were pelleted and lysed in RIPA buffer (50 mM Tris-HCl, pH 7.4, 150 mM NaCl, 20 mM EDTA, 1% triton X-100, 1% sodium deoxycholate and 0.1% SDS) supplemented with protease inhibitor cocktail “Complete Mini, EDTA free” (Roche, Meylan, France) and with 1/100 diluted phosphatase inhibitor (“Phosphatase Inhibitor cocktail II”, Sigma), as recommended by the suppliers. For immunoblotting experiments, the protocol conditions for using antibodies anti-Grb2, anti-actin and anti-GFP were as recently indicated [31]. Two specific antibodies were used to analyze SRSF5 expression: anti-SRSF5 polyclonal antibody (C-12; Santa Cruz Biotech., INC.), and the monoclonal antibody mAb104 [67], which defines the prototypical SRSFs by recognizing a common phosphoepitope in the C-terminal RS-domain. Polyclonal rabbit anti-CLK1 antibody (antibodies-online GmbH, Paris, France) was used to characterize CLK expression during erythroid differentiation. Anti-Grb2, mouse anti-α-tubulin (Sigma) and anti-actin served to standardize sample loading and protein integrity.

### Fluorescent microscopy

MEL cells expressing the fusion protein EGFP-SRSF5 were cultured on fibronectin-coated cover slips overnight at a starting confluency of 5 10^5^ cells/ml. Adherent cells were washed twice with 1×PBS, and fixed for 5 min at room temperature in 1 ml of fixation solution (0.4 g sucrose, 1 ml formaldehyde, 20 ml 1×PBS). Cells were then rinsed twice with 1×PBS and once with filtered sterile water. Fixed cells were stained with a DAPI solution (Invitrogen), as recommended by the manufacturer. The cover slips were incubated at 4°C in the dark, and fluorescence was apprehended by fluorescent microscopy.

### Bioinformatics sequence analysis

Sequence analysis and ESE motif search was performed using the ESEfinder web-based program (http://rulai.cshl.edu/tools/ESE2/).

## Supporting Information

Figure S1 **Decreased expression of SRSF5 during erythroid differentiation.** MEL cells were stably transfected with EGFP-SRSF5 construct and cultured in the absence (−) or presence (+) of DMSO for 4 days. Immunoblot analysis using mAB104 antibody reveals a dramatic and concomitant decrease of both endogenous SRSF5 and exogenous EGFP-SRSF5 in treated cells.
(TIF)

Figure S2 **SRSF5 knockdown and impact on pre-mRNA splicing in pre-differentiated MEL cells.** EGFP-SRSF5 cells were transfected with siRNA specifically targeting SRSF5 transcripts. SRSF5 mRNAs and proteins were analyzed to assess the knockdown efficiency 24 and 48 h after transfection. Mock cells were transfected with irrelevant siRNA.A. Real-time RT-PCR. SRSF5 mRNA derived from the endogenous gene and the stably-transfected EGFP-SRSF5 construct, were quantified by real-time RT-PCR using F7 and R7 primers (Table S1), and normalized to actin mRNA. SiSRSF5-mediated knockdown resulted in substantial mRNA decrease, as compared with mock cells.B. Immunoblot analysis. SRSF5 protein expression was estimated by western blot using mAb104 antibody and anti-GFP antibody. These experiments clearly showed that both the endogenous SRSF5 and fusion EGFP-SRSF5 proteins decreased specifically in cells treated with siSRSF5, while irrelevant siRNA had no effect in mock cells. Actin immunoblot served as control.C. Impact of SRSF5 knockdown on exon 16 splicing. Exon 16 inclusion was estimated by semi-quantitative RT-PCR on cells transfected with siSRSF5 or irrelevant siRNA (Mock). Exon inclusion remained very low within a range of 0–5%.
(TIF)

Table S1 **Primers used in this study.** Mismatches (underlined sequences) were introduced to disrupt the ESE within exon 16 (F1 and R1), a stop codon in EGFP-SRSF5-ΔRS construct (R3), or to mutate Ser86 residue (S86A-S and S86A-AS). Heterologous sequences were added in 5′ of some primers (bolded), to create restriction sites (italic) for cloning purposes. F: forward primers. R: reverse primers.
(DOCX)
