# Supplementary figures and images for: Proteasome-Mediated Proteolysis of SRSF5 Splicing Factor Intriguingly Co-occurs with SRSF5 mRNA Upregulation during Late Erythroid Differentiation
Source: PLoS One. 2013 Mar 11;8(3):e59137. doi: 10.1371/journal.pone.0059137 (PMC3594168; doi:10.1371/journal.pone.0059137)

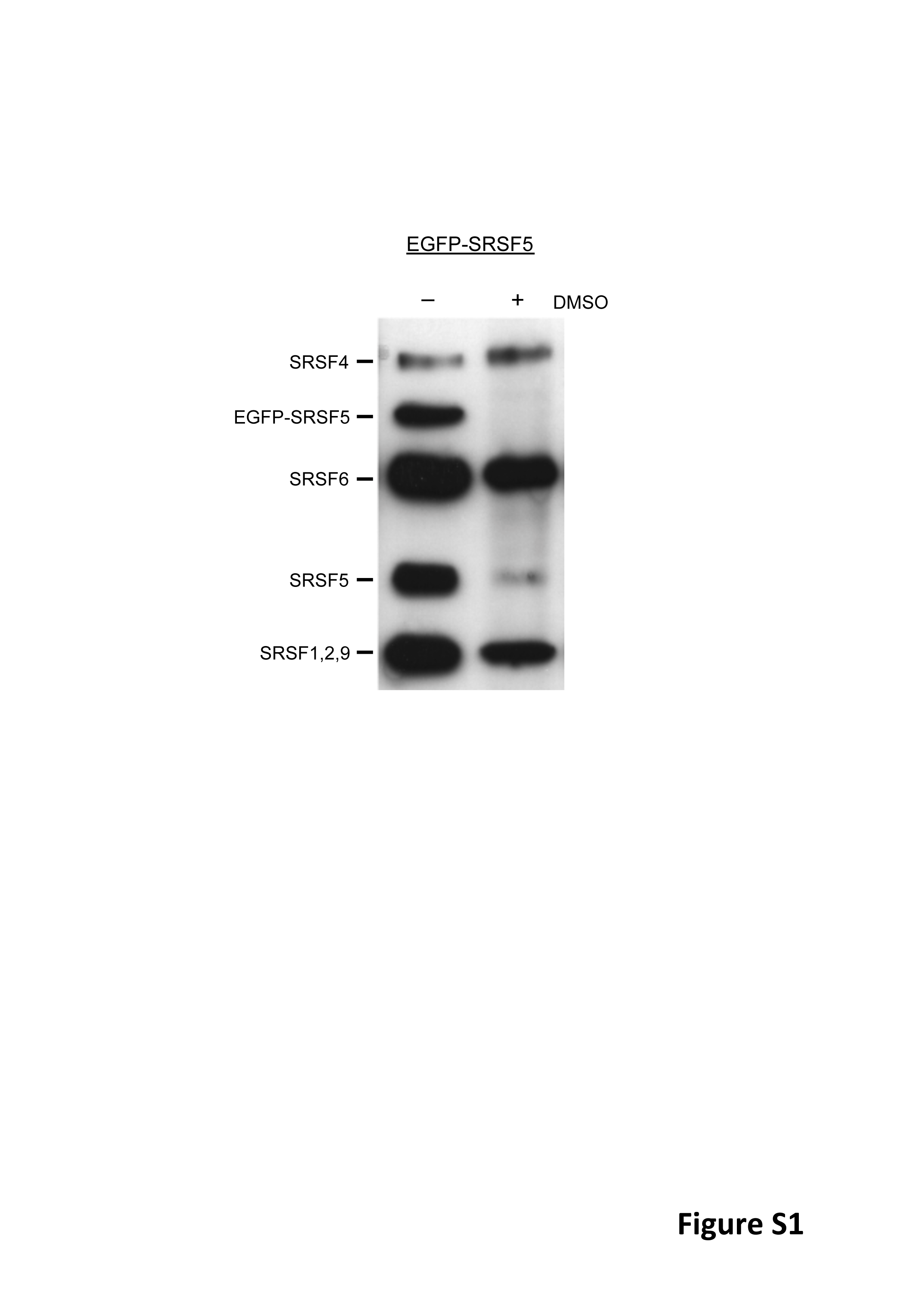

Supplement: Figure S1 — Decreased expression of SRSF5 during erythroid differentiation. MEL cells were stably transfected with EGFP-SRSF5 construct and cultured in the absence (−) or presence (+) of DMSO for 4 days. Immunoblot analysis using mAB104 antibody reveals a dramatic and concomitant decrease of both endogenous SRSF5 and exogenous EGFP-SRSF5 in treated cells. (TIF) [file pone.0059137.s001.tif]

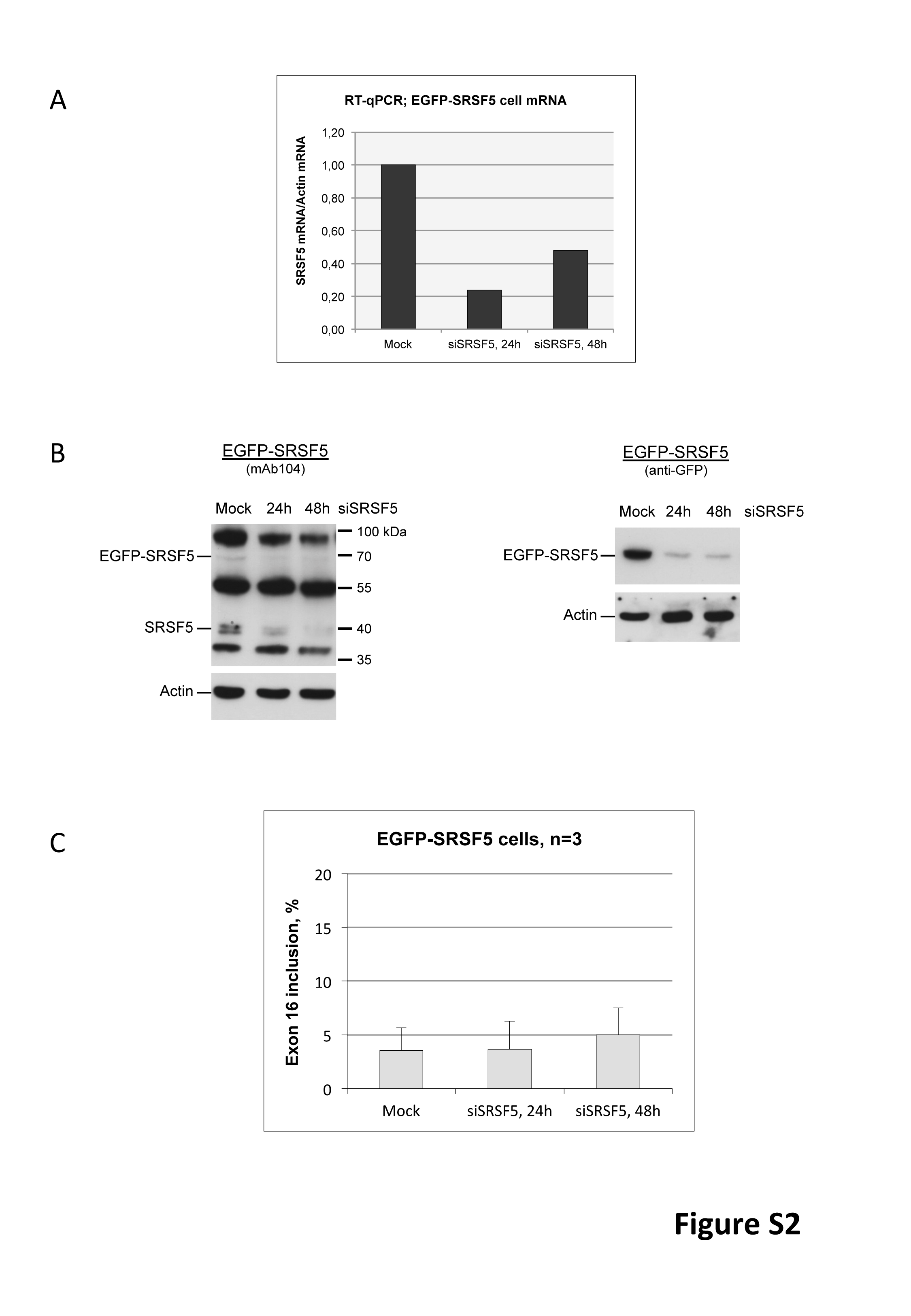

Supplement: Figure S2 — SRSF5 knockdown and impact on pre-mRNA splicing in pre-differentiated MEL cells. EGFP-SRSF5 cells were transfected with siRNA specifically targeting SRSF5 transcripts. SRSF5 mRNAs and proteins were analyzed to assess the knockdown efficiency 24 and 48 h after transfection. Mock cells were transfected with irrelevant siRNA.A. Real-time RT-PCR. SRSF5 mRNA derived from the endogenous gene and the stably-transfected EGFP-SRSF5 construct, were quantified by real-time RT-PCR using F7 and R7 primers (Table S1), and normalized to actin mRNA. SiSRSF5-mediated knockdown resulted in substantial mRNA decrease, as compared with mock cells.B. Immunoblot analysis. SRSF5 protein expression was estimated by western blot using mAb104 antibody and anti-GFP antibody. These experiments clearly showed that both the endogenous SRSF5 and fusion EGFP-SRSF5 proteins decreased specifically in cells treated with siSRSF5, while irrelevant siRNA had no effect in mock cells. Actin immunoblot served as control.C. Impact of SRSF5 knockdown on exon 16 splicing. Exon 16 inclusion was estimated by semi-quantitative RT-PCR on cells transfected with siSRSF5 or irrelevant siRNA (Mock). Exon inclusion remained very low within a range of 0–5%. (TIF) [file pone.0059137.s002.tif]
