# Supplementary material for: Proteasome-Mediated Proteolysis of SRSF5 Splicing Factor Intriguingly Co-occurs with SRSF5 mRNA Upregulation during Late Erythroid Differentiation
Source: PLoS One. 2013 Mar 11;8(3):e59137. doi: 10.1371/journal.pone.0059137 (PMC3594168; doi:10.1371/journal.pone.0059137)
Supplement: Table S1 — Primers used in this study. Mismatches (underlined sequences) were introduced to disrupt the ESE within exon 16 (F1 and R1), a stop codon in EGFP-SRSF5-ΔRS construct (R3), or to mutate Ser86 residue (S86A-S and S86A-AS). Heterologous sequences were added in 5′ of some primers (bolded), to create restriction sites (italic) for cloning purposes. F: forward primers. R: reverse primers. (DOCX) [file pone.0059137.s003.docx]

**Table S1**

| **Primer** | **Sequence (5'-3')** | **Location** |
| --- | --- | --- |
| F1 | AGAGAAAGACATGTTGGTGAAAAC | Mouse 4.1R exon 16 |
| R1 | TTTTCACCAACATGTCTTTCTCTC | Mouse 4.1R exon 16 |
| F2 | **CCG*TCCGGA***ATGAGTGGCTGTCGAGTGTTC | Mouse SRSF5 exon 2 |
| R2 | **CCG*GAATTC***TTAATTGCCACTGTCAACTGA | Mouse SRSF5 exon 8 |
| R3 | **CCG*GAATTC***TTAGTGCCTTTTGCTGCCTTC | Mouse SRSF5 exon 7 |
| F4 | **GA*GAATTC***GAAACACTAGCCGGGCATCA | Mouse SRSF5 exon 2 |
| R4 | **TT*GAATTC***AGTTTTAATTGCCACTGTC | Mouse SRSF5 exon 8 |
| F5 | **GG*GAATTC***GGAGCGTTAGGATTTGAG | Mouse SRSF3 exon 1 |
| R5 | **GT*GAATTC***CTGTACACCACTTTTGC | Mouse SRSF3 exon 6 |
| F6 | tgggaatgggtcagaaggactc | Mouse β-actin exon 3 |
| R6 | ctgggtcatcttttcacggttg | Mouse β-actin exon 3 |
| F7 | CGATTGAACATGCCCGGGCT | Mouse SRSF5 exon 4 |
| R7 | CTGCCAGCTGACTCTTGAGG | Mouse SRSF5 exon 5 |
| F8 | ATGGTGAGCAAGGGCGAGGA | EGFP |
| R8 | CTCCCTCGCTGCTGGATTTA | Mouse SRSF5 exon 2 |
| F9 | GCTTTTGGCTATTATGGACCAC | Mouse SRSF3 exon 2 |
| R9 | ACCATTCGACAGTTCCACTCTT | Mouse SRSF3 exon 3 |
| M13S | GTGAAGGTTGAAGAGAAGCGGG | Mouse 4.1R exon 13 |
| S86A-S | GAGGACGATACGCCGACCGTTTTA | Mouse SRSF5 exon 4 |
| S86A-AS | TAAAACGGTCGGCGTATCGTCCTC | Mouse SRSF5 exon 4 |
